# Correlation between metabolic syndrome and knee osteoarthritis: data from the Korean National Health and Nutrition Examination Survey (KNHANES)

**DOI:** 10.1186/1471-2458-13-603

**Published:** 2013-06-22

**Authors:** Chang Dong Han, Ik Hwan Yang, Woo Suk Lee, Yoo Jung Park, Kwan Kyu Park

**Affiliations:** 1 Department of Orthopaedic Surgery, Yonsei University College of Medicine, Seoul, Republic of Korea

**Keywords:** Metabolic syndrome, Knee, Osteoarthritis

## Abstract

**Background:**

This study was designed to investigate the correlations of knee osteoarthritis (OA) with metabolic syndrome (MetS) and MetS parameters in Korean subjects.

**Methods:**

This study included data from 270 subjects with knee OA and 1964 control subjects with a mean age of 54.56 (SD 11.53) years taken from the Korean National Health and Nutritional Examination Survey (KNHANES) 2008. Multivariate logistic regression analysis was conducted to examine possible associations for knee OA with MetS and MetS parameters.

**Results:**

MetS was shown to be associated with an increased risk of knee OA in female subjects in unadjusted analysis (OR 1.798, 95% CI 1.392, 2.322), but this significance disappeared when adjusted for confounding factors (OR 1.117, 95% CI 0.805, 1.550). No significant association between MetS and knee OA was found in male subjects. Among parameters of MetS, only high waist circumference (WC) in female subjects was significantly associated with an increased prevalence of knee OA, even after adjusting for confounding factors, while no other significant associations were found in both male and female subjects.

**Conclusion:**

We found that WC was associated with knee OA in female subjects, but neither MetS nor any parameters thereof were shown to be associated with knee OA in the Korean subjects of this study. Although we found no relationship between a pre-inflammatory state of MetS and knee OA, we believe further investigation of this relationship in various aspects is warranted, as MetS may also be a risk factor for complications in knee OA related procedures.

## Background

Osteoarthritis (OA) of the knee is a widely prevalent disease characterized by pain and limitations in daily activities caused by gradual deterioration and inflammation of the articular cartilages [1]. Of particular concern, the worldwide health and economic burden of knee OA will likely increase in the future, as longer life expectancy will lead to a growing elderly population [2]. The pathophysiologic mechanisms of OA are under debate, but there is general agreement that biomechanics and increased dynamic loading of the joint are involved [3]. However, it is suggested that other factors such as genetic, metabolic, and neuroendocrine factors may also contribute to increased incidences of knee OA [4].

Metabolic syndrome (MetS) comprises a number of conditions, including obesity, atherogenic dyslipidemia, impaired fasting glucose and hypertension (HTN), the prevalence of which has rapidly increased [5]. Nonetheless, a significantly increased risk of cardiovascular disease [6] and mortality [7] are reported among patients with metabolic syndrome. In musculoskeletal fields, MetS has gradually garnered greater attention due to an association with knee OA [8] and an increased risk of deep venous thrombosis after procedures related to knee OA, such as total knee arthroplasty (TKA) [9]. Obesity, a key factor in MetS, is well known to be associated with knee OA in terms of mechanical load, [10,11] and it is also thought to be related to excessive proinflammatory cytokine production which could play a pathophysiologic role in OA [12-16]. Furthermore, it has been also reported that atherogenic effects related to HTN could change the microvasculature of subchondral bone [17] and could have an effect on the development of knee OA. Nevertheless, studies concerning the synchronous effects of MetS and the parameters thereof on knee OA are limited [3,8].

Therefore, in this study, we attempted to determine the relationships between knee OA, MetS and MetS parameters through a population-based study. This study had two purposes: 1) to examine the relationship between MetS and knee OA and 2) to determine the associations between MetS parameters and knee OA.

## Methods

### Data source and subjects

**Figure 1:**
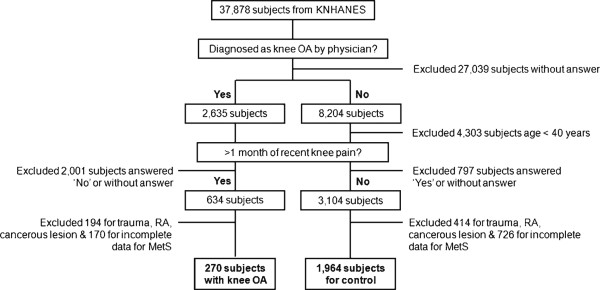
**Subjects included in the current study.** KNHSNES = Korean National Health and Nutrition Examination Survey; OA = osteoarthritis; M = month; RA = rheumatoid arthritis; MetS = metabolic syndrome.

Data were obtained from the Korean National Health and Nutrition Examination Survey (KNHANES) 2008. KNHANES was conducted by the Ministry of Health and Welfare [18]. A stratified multistage probability sampling design was used to generate representative data among non-institutionalized Korean civilians [18]. A flowchart for the inclusion of the subjects in this study is presented in Figure 1. Among 37,878 subjects that participated in KNHANES, 27,042 subjects who did not respond to the first question of “Have you ever been diagnosed as having knee OA by a physician?” were excluded, while 2,635 subjects who responded by saying ‘yes’ and 8,204 subjects who responded by saying ‘no’ were included in the study. In an attempt to include patients with recently-inflicted knee OA, of those 2,635 subjects who responded ‘yes’ to the previous question, 2,001 patients who either did not respond or responded by saying ‘no’ to the question of “Have you had knee pain for the past month or longer?” were excluded from this study; the remaining 634 subjects responded by saying ‘yes’. Among them, 194 subjects with a history of trauma, rheumatoid arthritis, or a non skin cancerous lesion and 170 with incomplete data for variables defining MetS were excluded from this study. Finally, 270 subjects were recruited as ‘subjects with knee OA’ and their mean age was 64.5 (SD 10.1) years old with a range of 40 to 90 years old. Of those 8,204 subjects who responded ‘no’ to the first question, 4,303 subjects with an age of less than forty years old were excluded in order to minimize age bias. Of those remaining 3,901 subjects, in an attempt to exclude those who had not been diagnosed as having knee OA, but had the possibility of having knee OA, this study excluded 797 subjects who replied ‘yes’ or who did not reply to the question of “Have you had knee pain for the past month or longer?” The remaining 3104 subjects were categorized as control subjects. Among them, except for 414 subjects with a history of trauma, rheumatoid arthritis, or non skin cancerous lesion and 726 subjects with incomplete data for variables defining MetS, 1,964 subjects were finally recruited as the control subjects. The mean age of this group was 53.2 (SD 11.0) years old with a range of 40 to 91 years old.

### Covariates

Data concerning clinical characteristics were collected by history taking, physical examinations and laboratory analyses. History taking included basic demographics, medical history, and lifestyle habits. Cigarette smoking was categorized as never smokers, ex-smokers and current smokers, and alcohol use was dichotomized as current users and nonusers. Physical examinations included measurements of blood pressure and measures of body mass. Blood pressure was measured using a mercury sphygmomanometer in a seated position after a 10-minute rest period. Two measurements were made for all subjects at 5-minute intervals. An average of two measurements was used for the data analyses. Weight was measured on a calibrated balance-beam scale and height was measured in an upright position using a stadiometer. BMI was calculated by dividing weight (Kg) by height^2^ (square meters). Waist circumference (WC) was measured at the midpoint between the bottom of the rib cage and the top of the lateral border of the iliac crest with full expiration. Blood samples were collected from subjects on the morning after an overnight fasting, and analyzed at a national central laboratory.

### Metabolic syndrome

The presence of MetS was defined using the National Cholesterol Education Program (NCEP ATPIII) [19]. The cut-offs established for Korean adults, as proposed by “The Korean Society for the Study of Obesity”, were adopted for the criterion of abdominal obesity [20]. The criterion for high glucose was adopted from the guidelines established by the American Diabetes Association [21]. Subjects with three of more of the following parameters were considered as having MetS: abdominal obesity (WC ≥90 cm in men and ≥80 cm in women); hypertriglyceridemia ≥150 mg/dl; low high density lipoprotein cholesterol (HDL – C) <40 mg/dl in men and <50 mg/dl in women; high blood pressure ≥130/85 mmHg or use of antihypertensive medication; or high fasting glucose ≥100 mg/dl or under treatment for diabetes.

### Statistics

Clinical characteristics among the subjects with knee OA and controls were compared by Student’s *t* test for continuous variables and Pearson’s Chi square test or Fisher’s exact test for categorical variables. Multivariate logistic regression analysis was conducted to evaluate relationships between knee OA with MetS itself or its parameters. WC was divided into sex-specific quartiles to examine its relationship with the prevalence of knee OA. Statistical analysis was performed with the SPSS for Windows statistical package (version 20.0, SPSS Inc., Chicago, IL), and all *P-*values < .05 were considered significant.

### Ethics

KNHANES 2008 was performed by the Ministry for Health, Welfare and Family Affairs (MIHWAF) of Korea and the Korea Centers for Disease Control and Prevention (KCDC) and was approved by the institutional review board of KCDC (Registration number: 2009-I3).

## Results

Clinical characteristics according to the presence or absence of knee OA are shown in Table 1. The prevalence of knee OA in this study was 12.1% (*n* = 270) in total, 4.0% (*n* = 41) in men and 18.9% (*n* = 229) in women. Compared with subjects without knee OA, those with knee OA had higher mean age, BMI, WC, SBP, and LDL - C, as well as a higher prevalence of smoking (current and ex-smoking); meanwhile, they had lower mean weight and height, as well as a lower prevalence of exercise and alcohol intake (Table 1). In women, a greater number of clinical characteristics were significantly different between subjects with and those without knee OA than in men (Table 1).

**Table 1:** **Clinical characteristics of subjects by presence or absence of knee OA (*n *=2234)**

	**All**		**Men**		**Women**	
	**No (*n*** **= 1964)**	**Yes (*n*** **= 270)**	**No (*n*** **= 979)**	**Yes (*n*** **= 41)**	**No (*n*** **= 985)**	**Yes (*n*** **= 229)**
Age (y)	53.2 ± 11.0‡	64.5 ± 10.1	53.8 ± 10.8‡	65.3 ± 10.5	52.6 ± 11.3‡	64.3 ± 10.0
Weight (kg)	62.0 ± 10.1‡	58.3 ± 9.5	66.6 ± 9.6§	63.9 ± 10.8	57.5 ± 8.5	57.3 ± 8.9
Height (cm)	161.2 ± 8.7‡	153.9 ± 7.6	167.4 ± 6.0*	165.2 ± 5.8	155.0 ± 6.1‡	151.8 ± 6.0
BMI (kg/m^2^)	23.8 ± 3.1‡	24.6 ± 3.3	23.7 ± 2.9	23.4 ± 3.6	23.9 ± 3.2‡	24.8 ± 3.2
WC (cm)	82.7 ± 8.7‡	85.0 ± 9.5	85.3 ± 7.9	85.9 ± 9.9	80.1 ± 8.7‡	84.8 ± 9.4
SBP (mmHg)	126.4 ± 20.2‡	133.2 ± 20.9	128.7 ± 19.3	132.8 ± 20.5	124.0 ±20.7‡	133.3 ± 21.0
DBP (mmHg)	79.5 ± 11.5	79.4 ± 12.0	81.7 ± 11.5*	77.0 ± 11.8	77.4 ± 11.2†	79.8 ± 12.1
Fasting glucose (mg/dL)	99.2 ± 18.2	99.9 ± 17.9	100.2 ± 18.6	99.5 ± 17.0	98.3 ± 17.7	100.0 ± 18.1
HDL - C (mg/dL)	45.5 ± 10.3	45.0 ± 10.2	163.4 ± 84.4	153.9 ± 80.5	47.5 ± 10.2†	45.5 ± 9.8
TG (mg/dL)	148.2 ± 79.7	149.7 ± 66.3	43.5 ± 10.0	42.6 ± 12.4	133.2 ± 71. 6†	149.0 ± 63.7
Exercise	594 (30.2)†	58 (21.6)	340 (34.7)	13 (31.7)	254 (25.8)§	45 (19.7)
Alcohol intake	1305 (66.4)‡	134 (49.6)	816 (83.4)	35 (85.4)	489 (49.6)§	99 (43.2)
Smoking						
Never	1131 (57.6)‡	209 (77.4)	210 (21.5)*	5 (12.2)	921 (93.5)§	204 (89.1)
Current	590 (30.0)	40 (14.8)	535 (54.6)	19 (46.3)	55 (5.6)	21 (9.2)
Ex-smoker	243 (12.4)	21 (7.8)	234 (23.9)	17 (41.5)	9 (0.9)	4 (1.7)

Abbreviations: *OA* osteoarthritis, *BMI* body mass index, *WC* waist circumference, *SBP* systolic blood pressure, *DBP* diastolic blood pressure, *HDL - C* high density lipoprotein cholesterol; *TG* triglyceride.

Values are presented with mean ± standard deviations or number (%).

*P*-values: Student’s *t* test & Chi-square test or Fisher’ exact test; .050 < ^§^*P* < .1, ^*^P < .050, ^†^*P* < .010, ^‡^*P* < .001.

**Figure 2:**
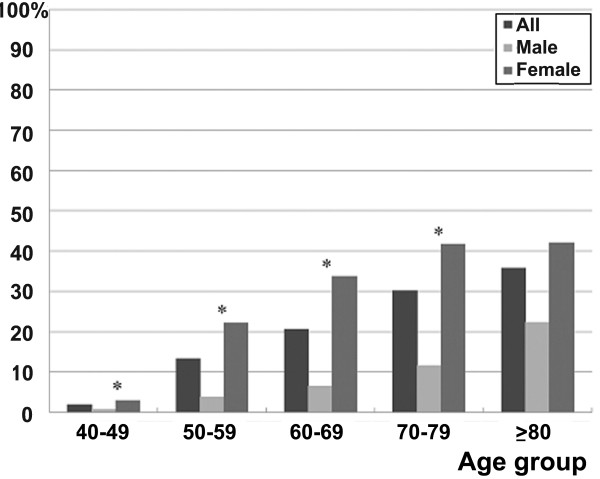
Prevalence of knee OA according to age groups. ^*^P value < .050 by Chi square test or Fisher’s exact test between male and female subjects. OA = osteoarthritis.

**Figure 3:**
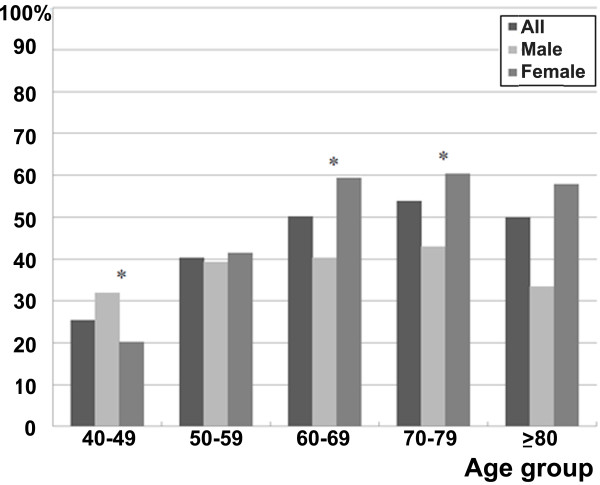
Prevalence of MetS according to age groups. ^*^P value < .050 by Chi square test or Fisher’s exact test between male and female subjects. MetS = metabolic syndrome.

The prevalences of knee OA and MetS, as well as the prevalence of MetS according to the presence or absence of knee OA, for individual age groups are shown in Figures 2 and 3 and Table 2. The prevalence rate of knee OA for patients in their 40s was 1.9% (0.7%, male; 2.9%, female), while that for patients in their 80s or older was 35.7% (22.2%, male; 42.1%, female), revealing an increasing prevalence for knee OA as patients became older (Figure 2). Except for patients in their 80s, the prevalence rate was significantly higher in females. In this study, the overall prevalence rate of MetS in all patients was 37.5% in males and 38.3% in females, showing a gradual increasing trend as one became older (Figure 3). Among those in their 40s, males exhibited a significantly higher prevalence rate of MetS, but the remaining age groups showed statistically similar or higher prevalence rates among females. In each age group, the prevalence of MetS was compared among patients with or without knee OA. However, there were no significant differences among overall patients, male or female (Table 2).

**Table 2:** Prevalence of MetS of subjects by presence or absence of knee OA

**Age (y)**	**All**			**Men**			**Women**		
	**Non - OA**	**OA**	***P***	**Non - OA**	**OA**	***P***	**Non - OA**	**OA**	***P***
40–49	244/945 (25.8%)	1/18 (5.6%)	.056	139/434 (32.0%)	0/3 (0%)	.555	105/511 (20.5%)	1/15 (6.7%)	.325
50–59	190/471 (40.3%)	29/72 (40.3%)	1.000	98/253 (38.7%)	5/10 (50.0%)	.520	92/218 (42.2%)	24/62 (38.7%)	.663
60–69	175/355 (49.3%)	49/92 (53.3%)	.559	82/202 (40.6%)	5/14 (35.7%)	.786	93/153 (60.8%)	44/78 (56.4%)	.572
70–79	78/157 (49.7%)	43/68 (63.2%)	.080	34/76 (44.7%)	3/10 (30.0%)	.504	44/81 (54.3%)	40/58 (69.0%)	.113
≥80	15/36 (41.7%)	13/20 (65.0%)	.162	4/14 (28.6%)	2/4 (50.0%)	.569	11/22 (50.0%)	11/16 (68.8%)	.326

Abbreviations: *MetS* metabolic syndrome, *OA* osteoarthritis.

Values are presented with number (%).

*P*-value: Chi square test or Fisher’s exact test.

MetS was associated with an increased risk of knee OA in female subjects prior to adjustment for potential confounding factors (OR 1.798, 95% CI 1.392, 2.322), but this significance disappeared when adjusted for age and sociographic factors of exercise, alcohol intake and smoking (OR 1.117, 95% CI 0.805, 1.550). No significant association between MetS and knee OA was found in male subjects prior to adjusting (OR 1.117, 95% CI 0.805, 1.550) or after adjusting for age and sociodemographic factors (OR 0.946, 95% CI 0.438, 1.852). The relationships of knee OA with parameters of MetS are shown in Table 3. Among parameters of MetS, only high waist circumference (WC) was significantly associated with an increased prevalence of knee OA in female subjects prior to adjusting (OR 2.004, 95% CI 1.464, 2.743), as well as after adjusting, for age and confounding factors (OR 1.838, 95% CI 1.311, 2.576); no other factors were found to be significant. No parameter of MetS was significantly associated with knee OA in male subjects (Table 3).

**Table 3:** ORs (95% CI) for knee OA according to parameters of MetS

	**Model 1**		**Model 2**	
	**Male**	**Female**	**Male**	**Female**
	**OR (95% CI)**	**OR (95% CI)**	**OR (95% CI)**	**OR (95% CI)**
WC (≥ 90/80 cm)	1.229 (0.615, 2.457)	2.004 (1.464, 2.743)	1.356 (0.650, 2.828)	1.838 (1.311, 2.576)
HTN	1.113 (0.586, 2.115)	2.000 (1.470, 2.722)	0.710 (0.361, 1.397)	0.933 (0.653, 1.334)
Hyperglycemia	1.334 (0.703, 2.533)	0.909 (0.670, 1.235)	1.458 (0.748, 2.839)	0.822 (0.591, 1.143)
Hypertriglyceridemia	0.798 (0.416, 1.530)	1.138 (0.827, 1.566)	0.997 (0.498, 1.999)	1.045 (0.742, 1.471)
Low HDL - C	1.202 (0.625, 2.311)	1.129 (0.816, 1.560)	1.020 (0.513, 2.027)	1.029 (0.724, 1.461)

Abbreviations: *OR* Odds ratio, *CI* confidence interval, *OA* osteoarthritis, *MetS* metabolic syndrome, *WC* waist circumference, *HTN* hypertension, *HDL - C* high density lipoprotein cholesterol.

Model 1: unadjusted. Model 2: adjusted for age, height exercise, alcohol intake, and smoking. ORs (95% CI) in Model 2 was 1.094 (1.060, 1.130) in male subjects and 1.089 (1.069, 1.108) in female subjects, and no significance was found with other factors.

In order to investigate the effect of WC on the prevalence of knee OA, WC was categorized into quartiles, and its relationships with knee OA was analyzed by multivariate logistic regression analyses (Table 4). The adjusted OR for knee OA with WC in the fourth quartile was 2.3 (1.144 – 3.546), when adjusted for age, exercise, alcohol intake, and smoking in women (Table 4).

**Table 4:** Prevalence of knee OA according to the quartile of waist circumference

	**Q1**		**Q2**		**Q3**		**Q4**	
	**Male**	**Female**	**Male**	**Female**	**Male**	**Female**	**Male**	**Female**
WC (Mean ± SDcm)	75.1 ± 4.2	69.7 ± 3.3	83.0 ± 1.4	77.8 ± 1.7	87.8 ± 1.4	83.4 ± 1.7	95.3 ± 4.6	92.9 ± 5.2
Prevalence of Knee OA	15/254 (5.9%)	31/302 (10.3%)	6/255 (0.6%)	46/304 (15.1%)	7/256 (2.7%)	66/304 (21.7%)	13/255 (5.1%)	86/304 (25.0%)
Adjusted OR^†^	1.0	1.0	0.8 (0.314-2.198)	1.3 (0.879-2.170)	0.4 (0.149-1.214)	1.7 (1.073-2.734)	0.9 (0.377-2.284)	2.3 (1.144-3.546)

Abbreviations: *OA* osteoarthritis, *WC* waist circumference, *Q* quartile, *OR* Odds ratio.

^†^ORs adjusted for age, sex, exercise, alcohol intake, and smoking. Adjusted ORs are presented with 95% Confidence Interval in parentheses.

## Discussion

This study investigated 1) relationships between MetS and knee OA, as well as 2) associations between each parameter of MetS and knee OA, via a population-based study. In this study, we found that only WC was associated with an increased risk of knee OA in female subjects.

The literature consensually agrees that the prevalence of MetS is increasing to a dangerous proportion [22-27]. The main concerns with the increasing prevalence of MetS are an increasing risk of diabetes [28] and cardiovascular disease [6]. As it affects knee OA, MetS is of particular concern for the following reasons: 1) obesity, a major key factor of MetS, has been shown to exert a direct mechanical effect on knee OA [10,11]; 2) in addition to the mechanical effect of obesity on knee OA, MetS as a pre-inflammatory condition might have a detrimental effect on the cartilages of the knee [29,30]; and 3) MetS potentially poses an increased risk of embolism in arthroplasty or other orthopaedic surgeries [9]. However, studies on the relationship between MetS and OA are limited [3,8]. Recently, Engström et al. [3] reported that MetS is associated with an increased incidence of knee OA when adjusted for age, sex and social factors in a Western population based study. They reported that this was largely explained by increased BMI; however, CRP was not associated with the incidence of knee OA after adjusting for confounding factors [3]. Gandhi et al. [8] reported that MetS is a risk factor for OA in Asians. In our study, MetS was not associated with an increased risk of knee OA after adjusting for confounding factors. A possible reason for the discordance between MetS and knee OA in this study could be that the knee OA subjects of this study may have had milder osteoarthritis than those of previous studies. Since the subjects with knee OA in previous studies included patients who had undergone total knee arthroplasty or high tibial osteotomy [3,8], the subjects might have had more severe knee OA than those of the current study.

Knee OA is thought to be associated with multifactorial causes [1]. There is a general consensus that knee OA is strongly associated with obesity. It has been reported that subjects with BMI >30 kg/m^2^ show a 4.2 − 6.8 times higher incidence of knee OA than control subjects [31,32]. In the current study, we also found that WC in female subjects was significantly related to knee OA. The reason for this is uncertain, but the prevalence of knee OA is known to be significantly higher in female subjects in Asian populations, [23,27,33-35] which was confirmed in this study as well. In addition to the mechanical effects of obesity or WC, knee OA is known to be closely related to inflammation and excessive production of proinflammatory cytokines, which are thought to play a role in the pathophysiology and disease progression of knee OA [12-15,36,37]. Serum concentrations of inflammatory markers, such as IL-6 and TNF-α have been shown to be higher in patients who have knee or hip OA [12-14], and are reported to be associated with increased radiographic progression of knee OA [13,15,37]. Diffusion of these cytokines from the synovial fluid into the cartilage is thought to contribute to cartilage matrix loss by stimulating chondrocyte catabolism and inhibiting anabolism in OA [11]. Furthermore, Singh et al. [38] reported that subjects with OA had a higher prevalence of DM and HTN, and proposed that pathology of small vessels in the subchondral bone could play a role in the development of OA. However, we found no significant correlations between the parameters of MetS, except for WC in female subjects, and knee OA in the current study. This may implicate that inflammatory pathways might not be correlated with knee OA; moreover, Onat et al. reported that there is discordance between insulin resistance and MetS [39]. However, we still believe that the relationship between MetS and knee OA deserves further study, because problematic complications such as deep vein thrombosis after procedures related to knee OA are known to be associated with MetS [9].

Several limitations should be noted in this study. First, the current study was a cross sectional study, which limited our ability to determine the causes and effects of MetS on knee OA. However, KNHANES is a representative sample of the general Korean population, and thus, our results could be generalized for the entire South Korean population. Second, there was a relatively small number of subjects with knee OA in this study, especially among males. This could explain why the prevalence of MetS was not significantly higher in subjects with knee OA than those without knee OA. Finally, identification of knee OA was derived mainly from a self-reporting manner, because KHANES did not include radiologic evaluation or physical examination for knee OA. We tried to exclude the subjects who may have been biased during sample selection, but we admit that our results could be different in knee OA subjects diagnosed by radiologic and physical examinations. Nevertheless, we showed that the prevalence of knee OA was consistent with other population based studies based on radiographs [40].

## Conclusions

We found that WC was associated with knee OA in female subjects, but neither MetS nor any parameters thereof were shown to be associated with knee OA in the Korean subjects of this study. Although we found no relationship between a pre-inflammatory state of MetS and knee OA, we believe further investigation of this relationship in various aspects is warranted, as MetS may also be a risk factor for complications in knee OA related procedures.

## Abbreviations

BMI: Body mass index; CI: Confidence interval; DBP: Diastolic blood pressure; HDL-C: High density lipoprotein cholesterol; HTN: Hypertension; KNHANES: Korean National Health and Nutritional Examination Survey; LDL-C: Low density lipoprotein cholesterol; M: Month; MetS: Metabolic syndrome; MIHWAF: Ministry for Health Welfare and Family Affairs; NCEP: National Cholesterol Education Program; OA: Osteoarthritis; OR: Odds ratio; Q: Quartile; RA: Rheumatoid arthritis; SD: Standard deviations; SBP: Systolic blood pressure; TG: Triglycerides; TKA: Total knee arthroplasty; WC: Waist circumference.

## Competing interests

The authors declare that they have no competing interests.

## Authors’ contributions

All authors took part in the planning and design of the study. CDH designed the study, YJP gathered the data, WSL analyzed the data, KKP wrote the initial drafts, and IHY ensured the accuracy of the data. All authors read and approved the final manuscript.

## Pre-publication history

The pre-publication history for this paper can be accessed here:

http://www.biomedcentral.com/1471-2458/13/603/prepub
